# Analytical electron microscopy analysis of insulating and metallic phases in nanostructured vanadium dioxide†

**DOI:** 10.1039/d4na00338a

**Published:** 2024-05-03

**Authors:** Jan Krpenský, Michal Horák, Jiří Kabát, Jakub Planer, Peter Kepič, Vlastimil Křápek, Andrea Konečná

**Affiliations:** a Institute of Physical Engineering, Brno University of Technology Technická 2896/2 616 69 Brno Czech Republic andrea.konecna@vutbr.cz krapek@vutbr.cz; b Central European Institute of Technology, Brno University of Technology Purkyňova 123 612 00 Brno Czech Republic

## Abstract

Vanadium dioxide (VO_2_) is a strongly correlated material that exhibits the insulator-to-metal transition (IMT) near room temperature, which makes it a promising candidate for applications in nanophotonics or optoelectronics. However, creating VO_2_ nanostructures with the desired functionality can be challenging due to microscopic inhomogeneities that can significantly impact the local optical and electronic properties. Thin lamellas, produced by focused ion beam milling from a homogeneous layer, provide a useful prototype for studying VO_2_ at the truly microscopic level using a scanning transmission electron microscope (STEM). High-resolution imaging is used to identify structural inhomogeneities while electron energy-loss spectroscopy (EELS) supported by statistical analysis helps to detect V_*x*_O_*y*_ stoichiometries with a reduced oxidation number of vanadium at the areas of thickness below 70 nm. On the other hand, the thicker areas are dominated by vanadium dioxide, where the signatures of the IMT are detected in both core-loss and low-loss EELS experiments with *in situ* heating. The experimental results are interpreted with *ab initio* and semi-classical calculations. This work shows that structural inhomogeneities such as pores and cracks present no harm to the desired optical properties of VO_2_ samples.

## Abstract 2

We utilize analytical electron microscopy with *in situ* heating to observe the insulator–metal transition in vanadium dioxide and to identify additional vanadium oxides across the sample exhibiting nanoscopic pores and cracks.

## Graphical abstract

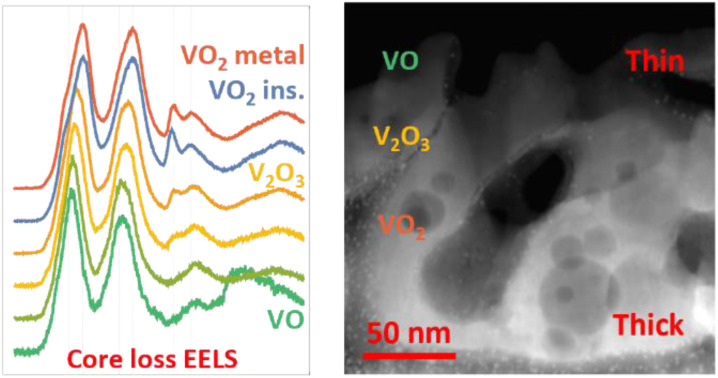

## 1 Introduction

Vanadium dioxide is a strongly correlated material that can undergo a volatile phase transition between the low-temperature monoclinic insulating phase and the high-temperature rutile metallic phase^1,2^ by an applied thermal,^3^ electrical,^4^ mechanical,^5^ or optical^6^ bias. In comparison to other phase-changing materials, VO_2_ stands out for its ultrafast (≈200 fs) IMT with a convenient transition temperature (≈67 °C) close to room temperature,^7^ which can withstand millions of switching cycles without degradation.^8^ All these properties suggest exciting applications of VO_2_ in sensing,^9^ energy storage,^10^ switching,^8,11,12^ data storage,^13,14^ or active optical metamaterials.^15–17^

The properties of the IMT in VO_2_ can be strongly influenced by crystallographic defects resulting from a substrate lattice mismatch and specific fabrication conditions.^5^ The IMT is also often dependent on VO_2_'s grain size, strain, and film thickness.^18–22^ Further complexity stems from the existence of multiple stable stoichiometries of vanadium oxide which can coexist within one sample or functional structure.^23–25^ Many of the applications of VO_2_ rely on nanostructuring, for which it is possible to use either top-down or bottom-up approaches.^26–29^ It is thus desirable to analyze the IMT and stoichiometry of vanadium oxide locally.

The IMT is tightly connected with an abrupt change in electronic structure, which determines electric and optical properties. Conventional ways to characterize the IMT thus involve measurements of the electric conductivity^30^ or optical response^19,31^ as functions of applied temperature. However, these methods have limited spatial resolution and do not allow the analysis of the IMT at the level of single nanostructures or even grains. One possible solution is represented by scanning near-field optical microscopy (SNOM), which helped to identify local inhomogeneities in the IMT with a spatial resolution of ∼10^1^ nm.^20,21,32–34^ Electron energy-loss spectroscopy (EELS) in a scanning transmission electron microscope (STEM)^35^ is another suitable technique allowing for spatial resolution down to single atoms, which can also be used to correlate the spectroscopic information with high-resolution imaging.^36,37^ Furthermore, EELS is particularly broadband and offers information on core-electron excitations with energies ∼10^2^ eV, optical excitations around ∼10^0^ eV and recently also vibrational excitations in the ∼10^−2^ eV range.^38^

EEL spectra in the core-loss region are dominated by excitations of electrons from core shells to unoccupied valence states. The spectral signatures are thus not only element-specific, but they also provide insights into local bonding, oxidation states of atoms,^39^ and geometrical confinement.^40^ Several studies have already exploited EELS near-edge fine structure to identify different stoichiometries of V_*x*_O_*y*_ samples^41–48^ or to study the hydrogenation of VO_2_.^49^ However, to the best of our knowledge, studies using (S)TEM in combination with EELS were up to one exception^31^ restricted to room temperature without the possibility of observing the changes when the material is switched to the high-temperature phase. Similarly, temperature-dependent low-loss STEM-EELS data that can be directly linked to optical properties are missing. In conducting materials, low-loss EELS is contributed by the excitation of plasmon resonances. This allows for a reliable distinguishing of conducting and insulating phases.

To gain insights into temperature-dependent properties of VO_2_ at the microscopic level, we study a lamella with gradually decreasing thickness fabricated from a thick evaporated layer of *presumably* VO_2_ by focused ion beam (FIB) lithography. The lamella is attached to a commercial heating chip, which allows for external control of the lamella temperature. The lamella is then thoroughly studied by analytical methods available in TEM. We use high-resolution imaging and STEM with energy-dispersive X-ray spectroscopy (EDX) to analyze the lamella structure and elemental composition. The optical and electronic properties of the lamella are characterized by temperature-dependent STEM-EELS. We complement our experimental observations with *ab initio* calculations of the core-loss probability. Low-loss probability is retrieved from the experimental dielectric function using a semi-classical formalism.^50^

## 2 Methods

### 2.1 Preparation of a thick VO_2_ layer

A 550 nm-thick VO_2_ layer was fabricated on a silicon substrate by evaporating VO_2_ powder (Mateck) in an electron beam evaporator (Bestec, 8 kV, 32 mA, 1 Å s^−1^) at room temperature and subsequent *ex situ* annealing for 20 min at 550 °C in a vacuum furnace under 10 sccm of O_2_ flow.

### 2.2 Preparation of the lamella

Lamellas for TEM were fabricated using a gallium FIB Tescan LYRA3. The final polishing and thinning of the V-shaped lamella denoted in the following as “IS” was performed with a low voltage (5 kV) and low current (40 pA) FIB for approximately 25 seconds at a low glancing angle (the ion beam is nearly parallel with the surface of the lamella) to reduce the beam-damaged surface area.^51^ The positioning of the lamella on the heating chip is shown in detail in Fig. S1 of the ESI.†

### 2.3 Transmission electron microscopy

TEM analysis was performed on a TEM FEI Titan equipped with a Super-X spectrometer for EDX, a monochromator, and a GIF-Quantum spectrometer for EELS. The heating experiment was performed using an *in situ* holder Fusion Select and the corresponding thermal chip by Protochips.^52^ To avoid excessive electron-beam-induced damage to the sample, we have utilized as low electron currents as possible while keeping a good signal-to-noise ratio. Further, we performed each step of the analysis in a new spot on the sample, so far unexposed. On the other hand, the distance between the spots was minimized to reduce the impact of the sample's inhomogeneity.

#### 2.3.1 STEM-EDX

EDX measurement was performed in the scanning regime with the energy of primary electrons set to 300 keV. The probe current was set to approximately 600 pA. We acquired and integrated 100 spectrum images with a pixel size of 0.34 nm and a dwell time of 2 μs. The spectrometer dispersion range was set to 20 keV. Chemical composition was evaluated in Velox software using the parabolic background model, absorption correction, and Schreiber–Wims ionization cross-section model (a semi-empirical model recommended for metal oxides). For the quantification of the vanadium dioxide layer stoichiometry, we used V K, O K, and Ga K edges.

#### 2.3.2 STEM-EELS core-loss

Core-loss EELS was performed in the monochromated scanning regime with the energy of primary electrons set to 300 keV. We used the following parameter settings: the probe current of around 200 pA, the convergence semi-angle of 10 mrad, the collection semi-angle of 18.4 mrad, and the spectrometer dispersion of 0.25 eV per px. The full-width at half-maximum of the zero-loss peak (determining the energy resolution) read 0.2 eV. We used the dualEELS option, which allows us to record both the low-loss and core-loss EELS spectra simultaneously, resulting in the correct calibration of the energy loss axis to absolute values.^53^ The spectrum line scans were acquired with a length of 100 pixels, a pixel size of 1.44 nm, and an exposure time of 0.1 ms for the low-loss part and 1 s for the core-loss part, respectively. The low-loss part of the spectrum was further used to determine the thickness profiles in Fig. 2(c) in the units of inelastic mean free path (IMFP).

#### 2.3.3 STEM-EELS low-loss

Low-loss EELS was performed in the monochromated scanning regime with the primary electron energy of 120 keV. The reduced value of the primary electron energy was used to achieve a better signal-to-background ratio^54^ and to suppress relativistic losses like the Čerenkov radiation.^55^ We used the following parameter settings: the probe current of approximately 100 pA, the convergence semi-angle of 10 mrad, the collection semi-angle of 11.4 mrad, and the spectrometer dispersion of 0.1 eV per pixel. The full-width at half-maximum of the zero-loss peak (determining the energy resolution) read 0.14 eV. We summed 100 spectra recorded over a 10 × 10 pixel spectrum image with a pixel size of 1 nm and an exposure time of 0.8 ms. The zero-loss peak subtraction was performed by a background power-law fitting respecting the shape of the pre-recorded zero-loss peak in a vacuum.

## 3 Results and discussion

### 3.1 Sample fabrication and pre-characterization

**Fig. 1:**
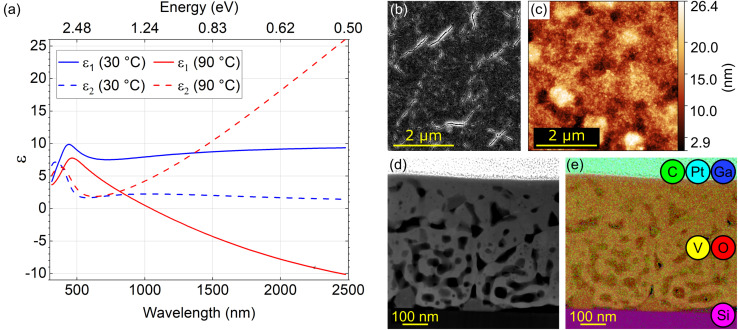
Pre-characterization of the VO_2_ thin film. (a) Optical properties below (blue) and above (red) the IMT temperature (≈67 °C). (b) Illustrative SEM image of the surface of the sample. (c) AFM image of the surface from a different place than (b). (d) STEM-HAADF image of a thin lamella “S” displaying a porous character of the VO_2_ layer. (e) STEM-HAADF image with overlaid EDX maps from the same place as (d) visualizing the presence of individual elements detected in the sample: Si substrate at the bottom (pink); V (yellow) and O (red) in the middle; a mixture of C (green), Pt (cyan), and Ga (dark blue) as components of the Pt protective coating at the top.

A layer of *presumably* vanadium dioxide (VO_2_) with a thickness of 550 nm was prepared by electron beam evaporation and subsequent *ex situ* annealing (see Methods). We first performed macroscopic characterization of the sample by spectroscopic ellipsometry to verify that it indeed corresponds to VO_2_ and that it exhibits the desired optical properties and their switching across the IMT temperature as reported in the literature.^19,56^Fig. 1(a) shows real (solid lines; *ε*_1_) and imaginary (dashed lines; *ε*_2_) parts of dielectric function at temperatures below (30 °C, blue) and above (90 °C, red) the IMT temperature. The high-temperature phase is metallic, with a negative *ε*_1_ function above the wavelength of 1100 nm, while the low-temperature phase is insulating, with positive *ε*_1_ in the whole inspected spectral region. The onset of interband electronic absorption is observed below about 500 nm, manifested by a peak in *ε*_2_. Above 500 nm, *ε*_2_ increases as ∼*λ* in the metallic phase as a consequence of the Drude-like response and remains low in the insulating phase. The IMT in VO_2_ associated with the observed qualitative change in its optical properties gives rise to a possible application of VO_2_ structures in active photonics.^17^

We further characterized the homogeneity of the layer surface using a scanning electron microscope (SEM) and an atomic force microscope (AFM) as shown in Fig. 1(b) and (c), respectively. The SEM image shows small cracks throughout the whole surface of the layer. The AFM image obtained at a different place on the surface also shows roughness and apparent pores in line with previous reports.^57^ The presence of inhomogeneities observable already at the surface triggered the need for a cross-sectional structural and chemical analysis using a TEM.

We prepared a standard TEM lamella (further labelled as “S” which stands for standard) by FIB. A high-angle annular dark-field (HAADF) image of the ∼120 nm thick lamella (representing a cross-section of the original VO_2_ layer) is shown in Fig. 1(d). The transverse profile of the layer exhibits pores and cracks noticeable also in the SEM and AFM images. The TEM image overlayed by a map of the elemental composition obtained by EDX is shown in Fig. 1(e). From the bottom up we identify the silicon (pink) substrate, vanadium (yellow) and oxygen (red) forming the layer itself and a protective layer – a mixture of carbon (green), platinum (cyan), and a minor contribution of implanted gallium (dark blue) prepared by focused ion beam induced deposition (FIBID). The EDX analysis of the VO_2_ layer reveals the elemental composition reading 32 ± 6 atomic percent of vanadium, 68 ± 8 atomic percent of oxygen, and 0.3 ± 0.1 atomic percent of gallium (only negligible contamination). The mean stoichiometry VO_2.1±0.4_ determined by EDX is consistent with the expected VO_2_ composition of the layer. Within porous areas of the sample [dark areas in Fig. 1(d)] we found increased oxygen amount with the elemental rates of (28 ± 5)% of vanadium and (72 ± 9)% of oxygen. As we discuss in Section S7 of the ESI,† the surplus oxygen corresponds to the contamination rather than the apparent mean stoichiometry V_2_O_5.1±1.1_ corresponding to V_2_O_5_.

**Fig. 2:**
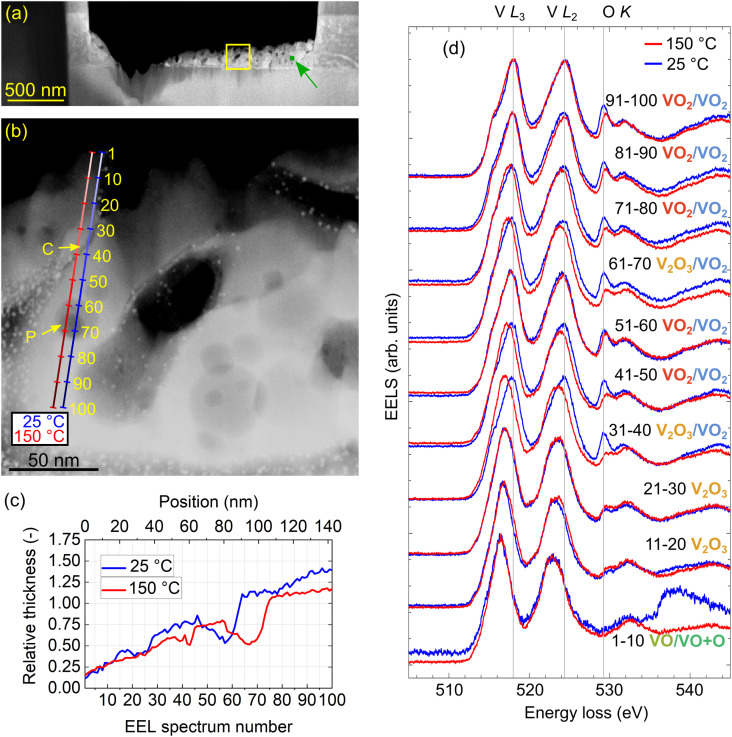
EEL spectrum line scans at nominal temperatures 25 °C and 150 °C. (a) STEM-ADF image of the lamella “IS”. Yellow-framed square marks the region of interest for the spectrum line scans, whereas the small green square, also denoted by the green arrow, will be used later for the low-loss EELS experiment. (b) Detail of the yellow-marked area. The blue line corresponds to a desired spectrum line scan at 25 °C, and the red line corresponds to a line spectrum image at 150 °C. Both spectrum line scans comprise 100 individual EELS measurements. Yellow arrows mark the crack (C) and pore (P). (c) Relative thickness in the units of IMFP, which varies around 130 nm for different V_*x*_O_*y*_ stoichiometries extracted from EELS. Note that the line scan at 25 °C slightly drifted from the desired position displayed in (b). (d) 2D waterfall graph with measured core-loss EEL spectra at both temperatures. Each spectrum averages ten subsequent EEL spectra as marked next to them. Vertical gray lines are to guide the eye.

To perform *in situ* analysis of the IMT in the TEM, another lamella (further labeled as lamella “IS” standing for *in situ*) was prepared and attached to a TEM heating chip (Protochips;^52^ see Fig. S1 of the ESI†). It consisted of a thick support and a thinned layer with gradually decreasing thickness dedicated for EELS measurements. Fig. 2(a) shows a STEM annular dark-field (ADF) image with an overview of the lamella “IS”. We further performed high-resolution imaging of the lamella and confirmed its polycrystallinity with random orientation of grains as shown in Fig. S2 of the ESI.† The yellow frame in Fig. 2(a) indicates the thinnest region of the lamella selected for further analysis.

### 3.2 Core-loss EELS

Our VO_2_ layer exhibits structural inhomogeneities, which might be associated with chemical inhomogeneities and irregularities in the switching from the insulating to the metallic phase. To explore this possible correlation, we analyze EELS of the lamella “IS” in the core-loss region. In Fig. 2(b), we show the target line scans along which we acquire 2 × 100 spectra with regular spacing. We perform the spectrum line scan close to room temperature (blue line) and above the IMT temperature (red line). The accuracy of the nominal temperature at the chip is reported to be 5% for a planar specimen positioned directly on the chip.^58^ However, the lamella is mostly free-standing and only connected to the heating chip by narrow pillars (see Fig. S1 of the ESI†) with presumably mediocre heat conductivity. Therefore, we assumed a larger discrepancy between the nominal and real temperature, and we heated the chip to 150 °C to ensure we were certainly above the IMT temperature. We also note that the designated line scan positions, especially the one for the measurement at 25 °C, slightly differ from the actual measurements due to sample drift. The thickness profiles extracted from EELS measurements in the units of the electron IMFP using the log-ratio method^35,59^ are shown in Fig. 2(c). The IMFP for our experimental parameters and possibly occurring different V_*x*_O_*y*_ stoichiometries varies around 130 nm (134 nm for VO_2_, 131 nm for V_2_O_3_ and 127 nm for VO based on the Mean free path estimator^60^ with model from ref. 61). Both line scans correspond to gradually increasing thickness with several valleys associated with the presence of the pores.

To facilitate visualization of the spectra and preliminary analysis, we binned the spectra into twenty groups by ten, and represented each bin by the average spectrum, which was further normalized by its maximum. The spectra shown in the waterfall plot in Fig. 2(d) globally exhibit up to five prominent distinct peaks. Notably, we can observe energy shifts and intensity changes in the peaks appearing in the spectra averaged over different beam positions. An additional feature, a pre-peak (a “shoulder”) of the first peak, is emerging in the spectra recorded in thicker areas. However, the changes in subsequent averaged spectra are rather abrupt, suggesting that a finer analysis is desirable.

**Fig. 3:**
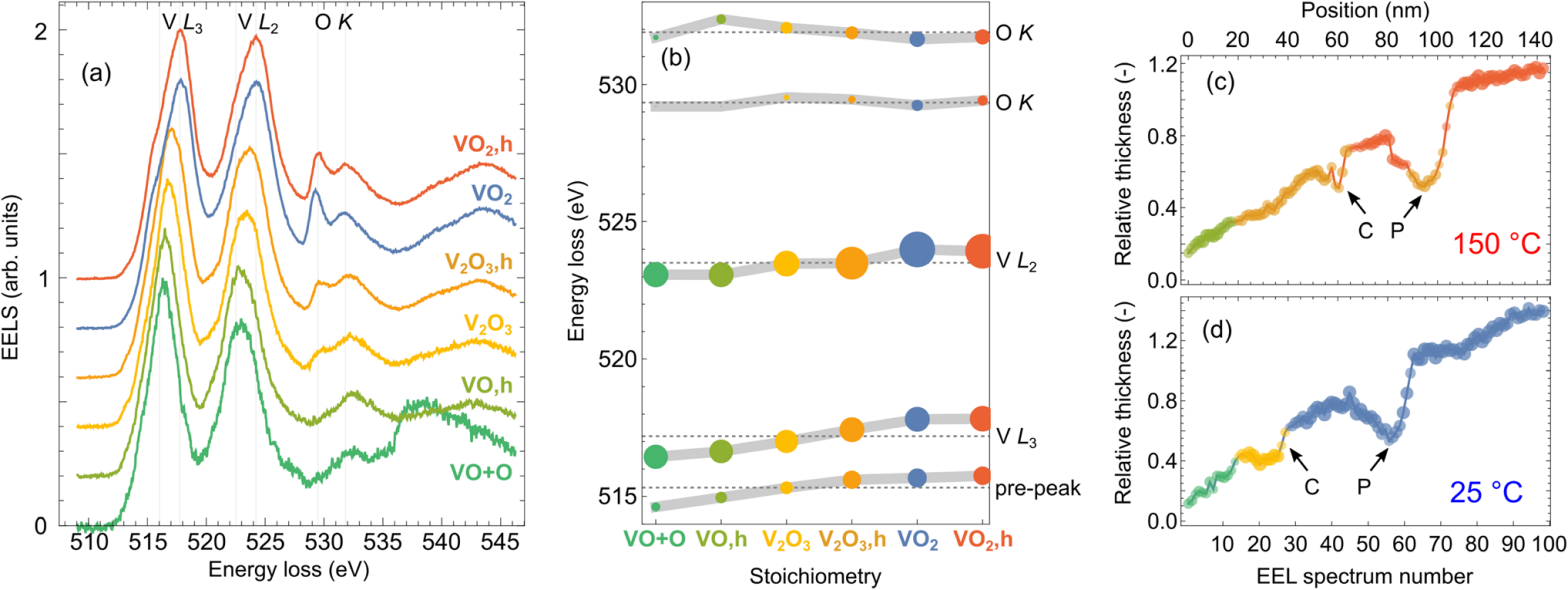
Cluster analysis of the spectrum line scans from Fig. 2. 100 spectra recorded within each spectrum line scan are sorted into three groups (clusters) according to similarities. The normalized averages of the spectra belonging to the individual clusters (cluster representants) are shown in (a). Fitting of the spectra in (a) by a sum of Gaussians yields peak energies plotted in (b), where the sizes of the dots are proportional to the square root of spectral areas of the peaks. Line profiles in (c and d) in the units of mean free path are overlaid by color-coded dots denoting affiliation of the spectrum recorded at the corresponding position to one of the clusters. The dots' size and opacity denote closeness to each cluster representant. The color-coding corresponds to (a). (c) is obtained for the measurement at 150 °C and (d) for 25 °C. Arrows mark the position of the crack (C) and the pore (P).

We utilize cluster analysis for a refined sorting of the acquired spectrum line scans. We sort each dataset (spectra acquired at room and elevated temperature, respectively) into clusters using the “*k*-means” clustering approach as implemented in Mathematica 12.^62^ Inspecting the results of several clustering procedures (with the number of clusters for each dataset varied between 2 and 4), we identified three clusters as optimum – all clusters were sufficiently populated and represented physically distinct spectra. Averaged spectra corresponding to the six acquired clusters, referred to as cluster representants, are plotted in Fig. 3(a).

The cluster representants exhibit multiple peaks, which can be assigned to transitions from the core to the unoccupied valence states of vanadium^63^ and oxygen:^41^ (i) V L_3_ peak (516–518 eV; transitions from 2p_3/2_ to the unoccupied 3d states of V), (ii) V L_2_ peak (523–524 eV; transitions from 2p_1/2_ to the unoccupied 3d states of V), and (iii) up to two O K peaks (529–532 eV; transitions of O electrons from 1s to hybridized O 2p–V 3d states, which are of t_2g_ and e_g_ symmetry). Spectral features observable above ∼535 eV arise due to transitions to O 2p and V 4s mixed states,^41^ but can be influenced by the presence of unbound oxygen or other oxygen compounds (*e.g.*, due to contamination^64^).

We further fit the cluster representants by a sum of seven Gaussian peaks (for details on fitting, see Fig. S3 of the ESI†). Energies of five of the fitted peaks corresponding to spectral features of our interest are plotted in Fig. 3(b). Notably, energies and relative strengths of V and O peaks strongly depend on the local oxidation state. The energies of V L_3_ and L_2_ peaks increase with a higher oxidation state of V, and importantly, we observe the emergence of a pre-peak, which has been attributed as a clear signature of crystalline VO_2_.^43,63^ Another clear signature is the increasing intensity of the first O K peak at ∼529 eV (V 3d t_2g_ bands).^41^ Such analysis allows for the precise assignment of our clusters with different V_*x*_O_*y*_ stoichiometries. We find that the first cluster in each dataset can be attributed to VO due to the missing first O K peak. However, we notice a difference between the cluster representants in the region around 539 eV. A broad peak associated with the O in other compounds or free O (ref. 64) appears for the case of the measurement at room temperature. We thus denote the cluster representant as “VO + O”. We then identify V_2_O_3_ (orange and yellow cluster representants) with two emergent O K peaks of comparable intensities. The last two cluster representants obtained for room and elevated temperature (blue and red spectra, respectively) exhibit a clear pre-peak around 515 eV and an intense first O K peak, which for both yields the VO_2_ assignment. However, a closer inspection reveals a slight energy shift and a different intensity ratio of O peaks between these two clusters. Interestingly, we did not identify any signature of V_2_O_5_, although it is rather common in nominally VO_2_ thin films.^57^ The EELS of V_2_O_5_ is typical by much higher contribution of the O K peaks compared to V L peaks.^65^

It is interesting to correlate the cluster index of each spectrum (*i.e.*, to what cluster an individual spectrum belongs) with the parameters at which the spectrum was taken: the position at the sample, the local thickness of the sample, and the temperature. We visualize this in Fig. 3(c) and (d). We represent each spectrum as a point in the sample-position–local-thickness coordinates, the color of the point represents the cluster index, and each panel corresponds to one of the two temperatures. Apparently, there is a strong correlation between the cluster index and the local thickness. On the other hand, as long as the thickness is identical, we do not observe significant differences between the crack, the pore and the homogeneous part of the lamella. Another significant parameter is the temperature. For each of the identified stoichiometries, the cluster representants at the room and the high temperature differ significantly, as shown in Fig. 3(b). We note that this difference is a mere indication of thermally dependent properties of all considered vanadium oxides (VO, V_2_O_3_ and VO_2_), but it does not provide evidence of the phase transition without further analysis.

The cluster analysis yields a following conclusion: With decreasing lamella thickness, we observe a gradual reduction of vanadium from VO_2_ to V_2_O_3_, and VO/VO + O. The part of the lamella with the reduced oxides is rather large, with a thickness up to 0.6–0.7 IMFP or 80–90 nm and a width (measured from the lamella edge) up to 60 nm [cmp. Fig. 3(c) and (d)]. This suggests difficulties in preparing ultrathin VO_2_ nanostructures, as the reduced oxides do not exhibit the desired switching of optical properties. On the other hand, a previous study reported considerably thinner (about 2 nm) diffuse layers of reduced vanadium oxides at the boundaries of individual VO_2_ grains,^43^ suggesting that with optimized fabrication protocols, the detrimental impact of the reduced oxides can be relieved.

Finally, we discuss the role of the structural inhomogeneity in the local properties of the lamella. The line scans shown in Fig. 2(b) cross two pronounced inhomogeneities: a narrow crack at the targeted spectrum indices of 35 (room temperature, blue line) and 40 (high temperature, red line) and a broad deep pore at the targeted spectrum index range of 60 to 70 for both temperatures. Both the crack and the pore are visible as depressions in the thickness profiles shown in Fig. 2(c). Here, the real spectrum indices slightly differ from the targeted ones due to sample drift, which resulted in an offset of 10 between the targeted and real spectrum indices for room temperature. The crack is found at the spectrum index of 22 (room temperature) and 43 (high temperature), and the pore is located in the spectrum index ranges of 46 to 64 (room temperature) and 58 to 75 (high temperature).

For the crack at both temperatures and for the pore at room temperature we observe no modification of the core-loss EEL spectrum in comparison to the areas of the lamella adjacent to the inhomogeneity, neither by visual inspection [Fig. 2(d)] nor by cluster analysis represented by the spectrum cluster index [Fig. 3(d)]. On the other hand, the core-loss EEL spectrum is modified for the deepest part of the pore at the high temperature, where the stoichiometry changes from VO_2_ to V_2_O_3_ [see Fig. 3(c)]. A closer inspection suggests that this modification is related to a reduced thickness of the lamella within the pore. In other words, the singular parameter determining the local electronic and optical properties of the lamella manifested in core-loss EELS is its thickness. The structural inhomogeneities (cracks and pores) themselves do not impose any observable modifications (*e.g.* due to their elastic or plastic field, different local composition, stoichiometry, or local charge) unless they are accompanied by a modified local thickness. As an important consequence, the chemical composition and stoichiometry of the vanadium oxide inside cracks and pores do not differ from those far from the cracks and pores (for the same local thickness). This also means that the presence of cracks and pores is not detrimental to the optical properties of the vanadium dioxide layer (while we cannot exclude an impact on its mechanical or transport properties).

We note that the area of the lamella identified as the crack is present in the region of V_2_O_3_ at the interface with VO_2_. Further investigation is required to determine whether this correlation is causal or coincidental, and possibly determine its mechanism.

### 3.3 Variation of core-loss and low-loss EELS with temperature

To verify that the temperature-dependent changes in our core-loss EELS measurements of VO_2_ are due to the IMT, we perform *ab initio* calculations. We use the TELNES3 package, the extension of the Wien2k all-electron DFT code,^66^ to simulate electron loss near-edge structure (ELNES) of the O K-edge. More details about the calculation setup are given in the ESI.†

**Fig. 4:**
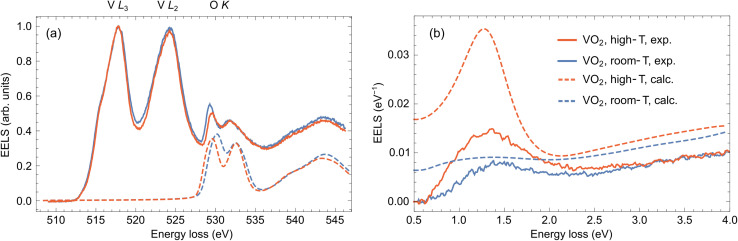
(a) Comparison of the averaged clustered spectra (cluster representants) assigned as VO_2_ obtained at 25 °C (solid blue line), and 150 °C (solid red line) with *ab initio*-calculated O K peaks corresponding to the monoclinic phase (dashed blue line) and rutile phase (dashed red line). The calculated spectra are both offset by 528.5 eV to get an approximate alignment with the experimental peak positions. (b) Comparison of the experimentally acquired low-loss EELS at 25 °C (solid blue line), and 150 °C (solid red line) from the area marked by the small green square and arrow in Fig. 2(a) compared with calculations using a classical dielectric formalism (dashed blue and red lines).

In Fig. 4(a), we present a comparison between the cluster representants of core-loss EELS obtained in the thick areas of the sample at 25 °C and 150 °C (solid lines) together with the calculated spectra (dashed lines; after convolution with 1 eV Gaussian) concerning the O K excitations. The *ab initio* theory predicts changes in the positions and relative strengths of the convolved O K peaks, the latter in qualitative agreement with our experimental observation. In line with the previous work of Hébert *et al.*,^41^ the relative intensity of the O K peaks is proportional to the number of O(p) states in the t_2g_ band (π-bonds; first O K peak) and in the e_g_ band (σ-bonds; second O K peak). Indeed, the ratio of O(p) states in respective bands changes from 80.7% to 77.4% after IMT, which explains a relative decrease of the first O K peak with respect to the second O K peak at elevated temperature.

Modifications in the band structure across the IMT are also tightly connected with changes in valence-electron excitations, which can be probed by EELS in the low-loss region. In the case of VO_2_, the IMT could be clearly identified based on the emergence of plasmons in the rutile (metallic) phase. Plasmons in VO_2_ nanostructures were previously detected only with high-resolution EELS,^67,68^ where slow electrons in a broad beam were used to probe the sample surface. However, to the best of our knowledge, low-loss STEM-EELS of nanostructured VO_2_ samples probed across the IMT have been missing.

Unfortunately, the long time necessary to acquire the core-loss EELS prevented us from performing the low-loss EELS experiment in the previously analyzed area due to accumulated contamination and beam-induced damage. We therefore collected the low-loss EEL spectra in the region close by, denoted by the green square in Fig. 2(a). This region is sufficiently thick (∼300 nm based on STEM-HAADF images of the lamella and a comparison of intensities with areas of known thickness) without too many pores, so it contains dominantly VO_2_. To avoid beam-induced damage, we collected a spectral image with 10 × 10 pixels with regular steps of ∼1 nm and averaged them. We further fitted and subtracted the zero-loss peak to obtain the results in Fig. 4(b) (solid lines; see raw spectra in Fig. S4 of the ESI†). The experiment was again performed below and above the IMT temperature, at 25 °C (blue) and 150 °C (red), respectively.

The experimental low-loss EEL spectra in Fig. 4(b) exhibit a broad peak between 1.0 and 1.5 eV, which grows in intensity when the sample is heated up. To interpret this behavior, we perform semi-analytical calculations within the framework of classical electrodynamics. To that end, we consider the problem of a line current (representing a focused electron beam) traversing a thin film characterized by a dielectric function from Fig. 1(a) and solve for the induced electric field entering the formula for electron energy-loss probability.^50^ The spectrum calculated for VO_2_ in the monoclinic (insulating) phase (dashed blue line) shows only a faint broad peak centered at ∼1.25 eV corresponding to weak absorption contribution, a small portion of radiative losses and an increasing background above 2.5 eV due to higher-energy interband transitions. On the other hand, the intense peak around 1.3 eV appears in the calculation for the rutile (metallic) phase (dashed red line), which can be assigned to bulk plasmon and surface plasmon polariton excitations. These theoretical results qualitatively support the experimental observation and confirm that the spectral change could be interpreted as IMT-induced. However, the experimentally measured loss probability is less intense, which is particularly apparent in the plasmonic peak in the spectrum recorded at 150 °C. Such quantitative discrepancy can happen due to differences in the actual and model dielectric responses of the sampled area, or partially because of the background subtraction procedure, where zero signal is assumed below 0.5 eV. Multiple scattering, which is not included in the theory, could also play some role.

## 4 Conclusions

We have studied the insulating and metallic phases of nanostructured vanadium dioxide (VO_2_) using analytical electron microscopy. We have focused on ultrathin lamellas fabricated from a 550 nm-thick VO_2_ film. With the help of STEM-EELS, we have revealed that the lamellas show both structural and stoichiometric inhomogeneities at the microscopic level. Results from the core-loss EELS enhanced by statistical analysis helped to correlate the stoichiometry with the thickness of the lamella, finding vanadium oxides with a reduced oxidation number (V_2_O_3_ and VO) at thinner parts of the lamella (with the thickness of 60 nm or less). This represents a challenge for preparing ultrathin VO_2_ nanostructures.

The principal contribution of our study is the observation of temperature-induced changes in both core-loss and low-loss EELS in thicker regions of the lamella, which were identified to contain dominantly VO_2_. Changes in the core-loss spectra were analyzed with the help of *ab initio* calculations that were used to relate the fine structure of the oxygen K peaks with the modifications in the band structure during the IMT. In the low-loss spectra, the most significant evidence of the IMT is the emergence of a strong plasmonic peak in the metallic phase.

This work establishes the STEM-EELS with *in situ* heating as a suitable technique to probe the IMT in VO_2_, and potentially in other materials. We foresee that STEM-EELS could also be combined with *in situ* diffraction studies to correlate the spectroscopic results with crystal structure and orientation to further enhance the analysis of the IMT.

## Conflicts of interest

There are no conflicts to declare.

## Supplementary Material

NA-006-D4NA00338A-s001
