# Supplementary material for: Analytical electron microscopy analysis of insulating and metallic phases in nanostructured vanadium dioxide
Source: Nanoscale Adv. 2024 May 3;6(13):3338–46. doi: 10.1039/d4na00338a (PMC11197434; doi:10.1039/d4na00338a)
Supplement: NA-006-D4NA00338A-s001 [file NA-006-D4NA00338A-s001.pdf]

Electronic Supplementary Material (ESI) for Nanoscale Advances.
This journal is © The Royal Society of Chemistry 2024

In-situ electron energy-loss spectroscopy of inhomogeneous ultrathin vanadium dioxide

– SUPPLEMENTARY INFORMATION –

Jan Krpenský,[1] Michal Horák,[1,2] Jiří Kabát,[1] Jakub Planer,[2]
Peter Kepič,[1,2] Vlastimil Křápek,[1,2] and Andrea Konečná[1,2]

[1] *Institute of Physical Engineering, Brno University of Technology, Technická 2896/2, 616 69 Brno, Czech Republic*
[2] *Central European Institute of Technology, Brno University of Technology, 61200 Brno, Czech Republic*

S1. PREPARATION OF THE LAMELLA ON THE HEATING CHIP

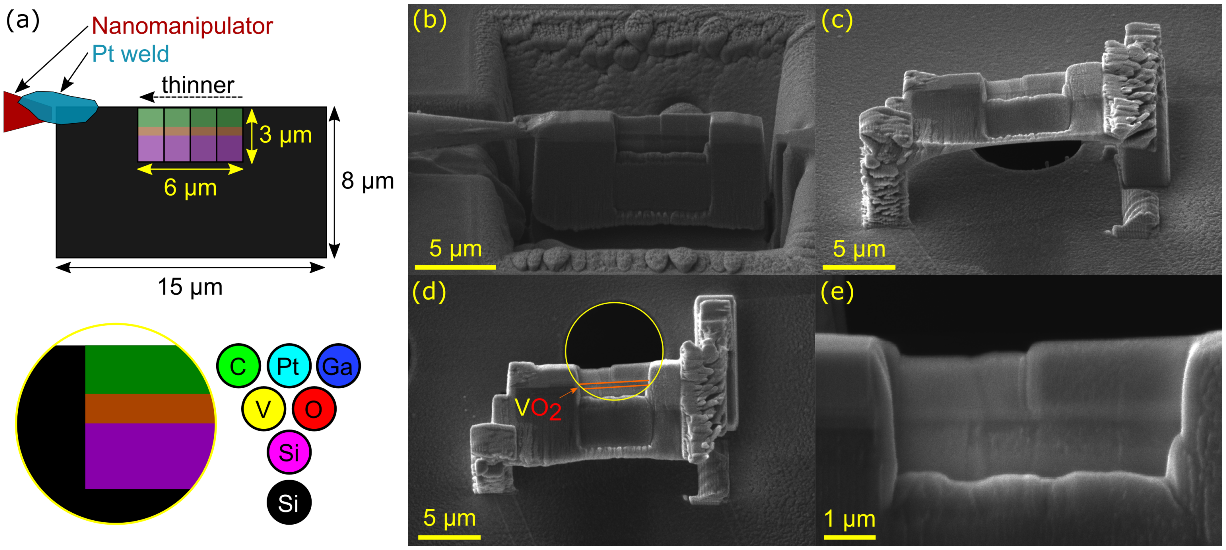

FIG. S1. Fabrication of lamella "IS" for further TEM analysis. (a) Schematic drawing showing dimensions (top) and chemical composition (bottom) of the lamella. Black colored Si used as a support frame for the lamella is much thicker (>1 µm) than purple colored Si (<200 nm). The colorful lamella window ($3{\times}6\,\mu m^2$) gets thinner from right to left. (b) SEM image of the lamella schematically shown in (a). (c) Lamella placement on a heating chip – SEM image at 35° angle. (d) Top view of the lamella showing a hole in the heating chip (yellow circle) and VO_2 layer (orange lines). (e) Detail of the thinned area used for further analysis.

Fig. S1(a) shows the dimensions and chemical composition of the lamella "IS" [color coding agrees with Fig. 1(e) of the main text]. The lamella "IS" is approximately three times larger than the standard lamella "S" analyzed in Fig. 1(d,e) of the main text and has a thick frame that was later used during its attachment to the heating chip. The challenging fabrication process of the lamella on the heating chip is shown in Fig. S1(b)-(d): (b) carving the lamella from the sample and lift-off with a nanomanipulator, (c) attachment of the lamella to the heating chip above one of the holes in the chip, so that (d) the VO_2 layer is directly above the hole. Fig. S1(e) shows a detail of the part of the lamella that is used for the subsequent TEM analysis. This area is divided into four parts, with their thickness decreasing from right to left, and in the following, we only show results for the two thinnest parts on the left. As later shown in Fig. S2(a), the protective layer (green) was removed, and the VO_2 layer was further thinned to achieve a thickness gradient from extremely thin near the vacuum (upper part) to thicker near the Si substrate (bottom part).

S2. HIGH-RESOLUTION IMAGING OF THE LAMELLA

In Fig. S2(a) we show an overview of the neighborhood of the analyzed part of the thinned lamella and a detail of the selected part of the analysis in (b). The lamella again shows inhomogeneities (pores and cracks) similar to the standard lamella "S" in Figure 1(d,e) of the main text. To reveal whether the thinned lamella is crystalline or not, we performed high-resolution imaging, as shown in Fig. S2(c). Fast Fourier transform (FFT) of the two areas assigned as "beach" (B) and "hill" (H) shown in (d) and (e), confirms that the lamella is crystalline, with random orientation

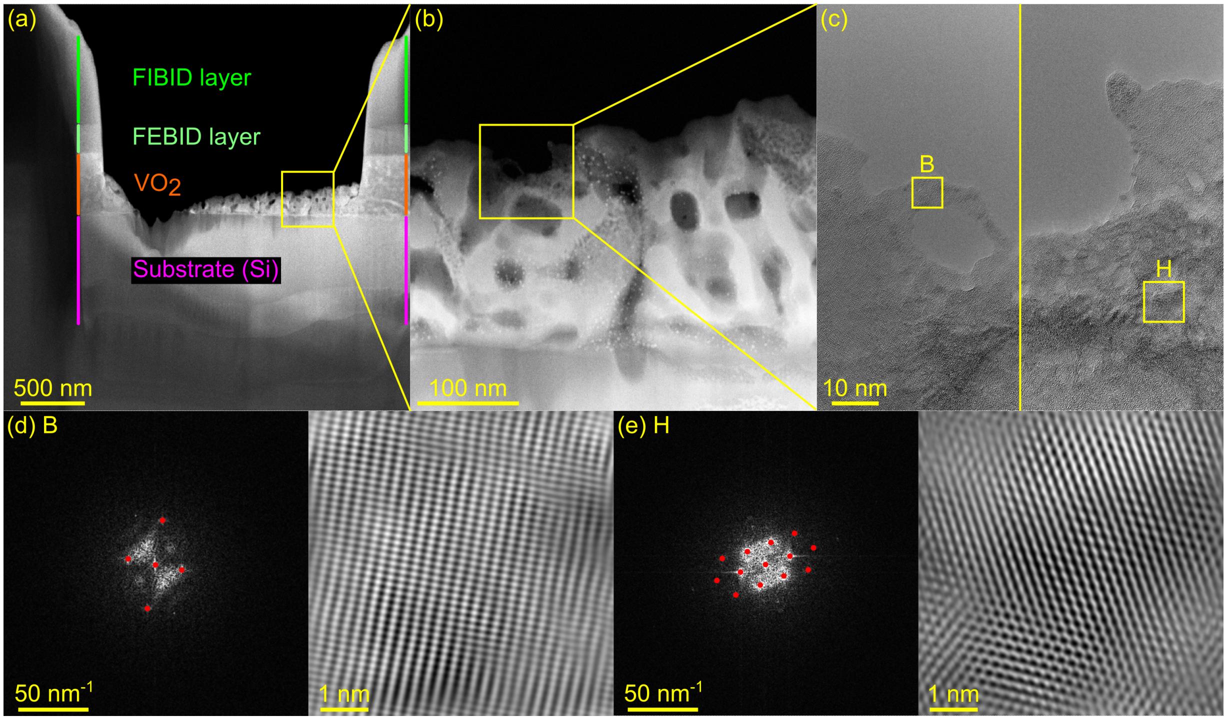

FIG. S2. High-resolution imaging of the thinned-down lamella. (a) Removal of protective Pt layer and thinning of VO_2 layer (STEM-HAADF image). The smaller protective layer of Pt near VO_2 layer was obtained by focused electron beam-induced deposition (FEBID) to minimize Ga implantation in VO_2, and the bigger part farther from VO_2 layer was made by focused ion beam-induced deposition (FIBID). (b) STEM-HAADF image of the detail in (a) showing porous VO_2 layer (brighter shades) against vacuum (black). (c) High-resolution TEM image of the detail in (b). The image is a combination of 2 individual images of the same area each with a different focus (divided by a vertical yellow line). Two areas beach (B) and hill (H) are marked. (d) (left) FFT of the beach area B where red dots correspond to discrete spatial frequencies in k-space; (right) inverse FFT created from marked spots. (e) (left) FFT of the hill area H where red dots correspond to discrete spatial frequencies in k-space; (right) inverse FFT created from marked spots.

of different grains. The left parts of the panels (d,e) show the Fourier transform of the selected areas while the right images are back-transformed images where we only filtered spatial frequencies around the brightest points assigned by red dots.

S3. FITTING OF CORE-LOSS EEL SPECTRA

We fit the spectra by a sum of seven Gaussian peaks, *i.e.*, we use a formula

$$\Gamma(E) = \sum_{i=1}^{7} a_i \exp\left[-\frac{(E-b_i)^2}{c_i^2}\right], \tag{1}$$

where a_i, b_i, and c_i are free parameters proportional to scaling, peak center, and width, respectively. For each of the two clearly visible vanadium peaks, V L_3 and L_2, we fitted one main peak and one pre-peak, *e.g.*, four peaks in total. Two peaks were used for fitting the region of the O K edge. The last peak, rather phenomenological, represented contributions at higher energies. The individual Gaussian functions contributing to the fits of core-loss EEL spectra for different cluster representants are plotted in Figure S3 in gray.

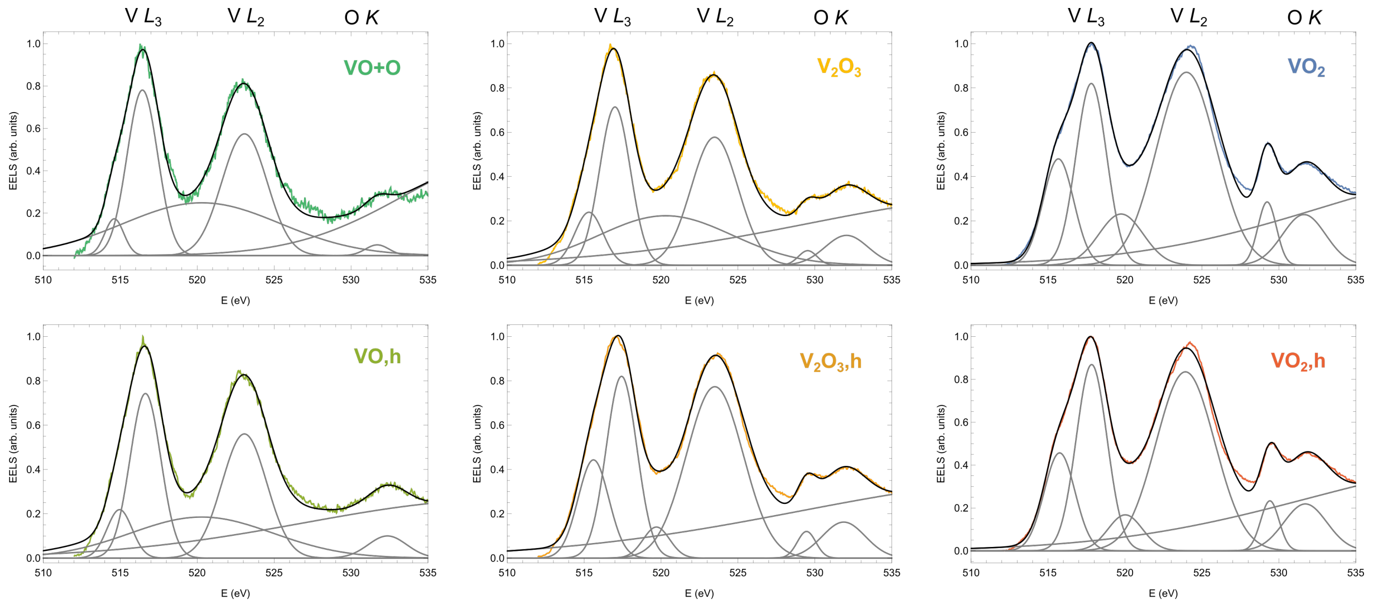

FIG. S3. Each panel shows the result of the fitting (black line) of the measured core-loss EEL spectrum (lines with colors corresponding to those in Fig. 3 of the main text) for a selected cluster representant. The seven individual Gaussian functions contributing to the fit are plotted by gray.

S4. FITTING CORE-LOSS EEL SPECTRA BEFORE CLUSTERING

In Fig. S4, we plot averaged couples of EEL spectra (*i.e.*, every two subsequent spectra were averaged to reduce the noise) along each linescan at room and high temperature (left and right panel, respectively). Before the clustering procedure, we fitted these averaged couples of spectra one by one to verify the conclusions obtained from clustering. By using the same fitting procedure as described above, we extracted positions (energies) and areas of selected fitted peaks: pre-peak of the V $L3$ peak, V $L3$ peak itself, V $L2$ peak and two O K peaks. The increase in energy of the vanadium peaks as a function of EEL spectrum number and the corresponding increasing thickness can be clearly observed in Fig. S5. We can also notice an increase in intensity of the pre-peak as well as of O K peaks.

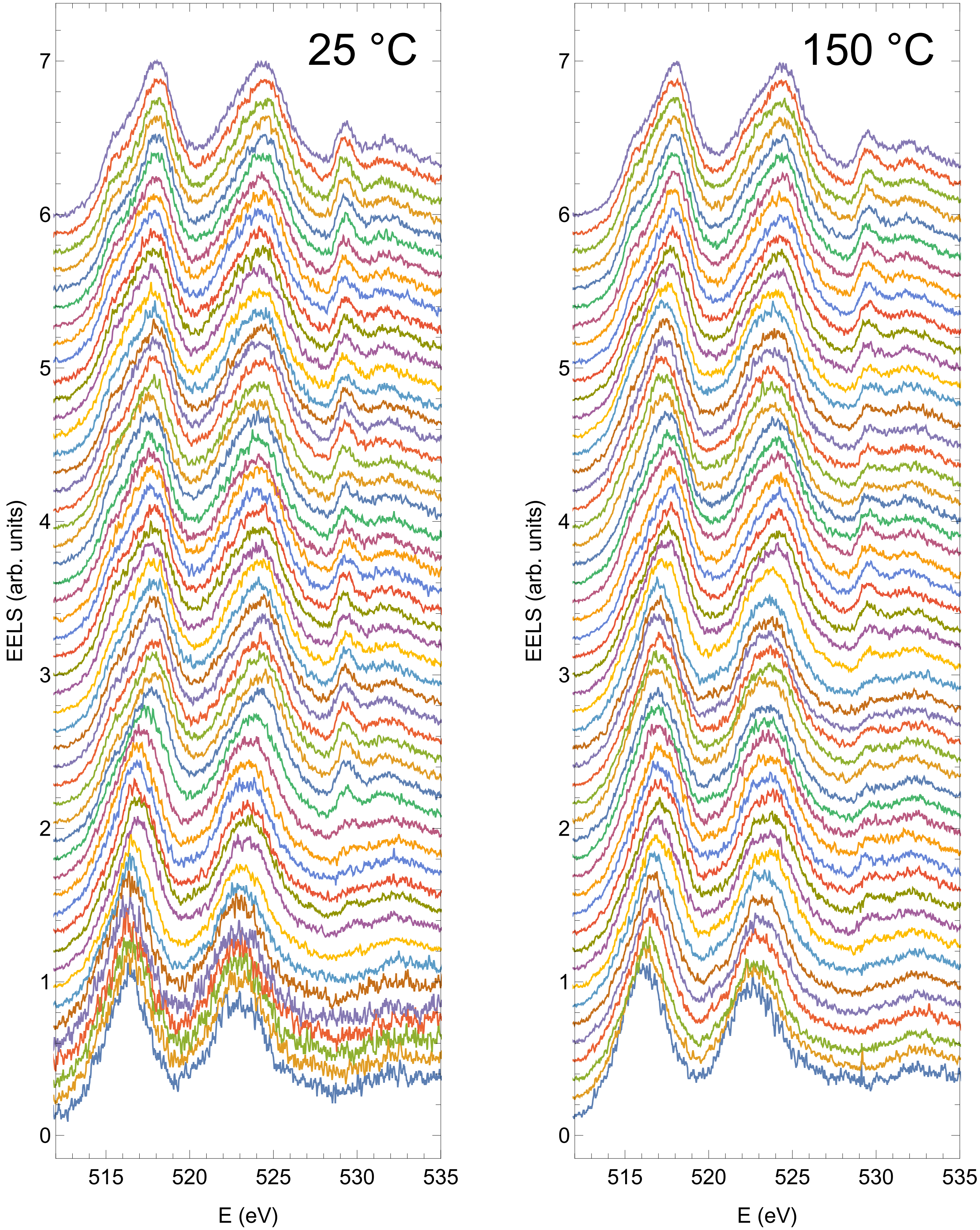

FIG. S4. Core-loss EEL spectra acquired along the linescans as described in Fig. 2 of the main text. Left/right panel corresponds to the spectra measured at room/high temperature. In each panel, we group the 100 spectra in couples and average over them, i.e. we plot 50 spectra in each panel, using a constant offset.

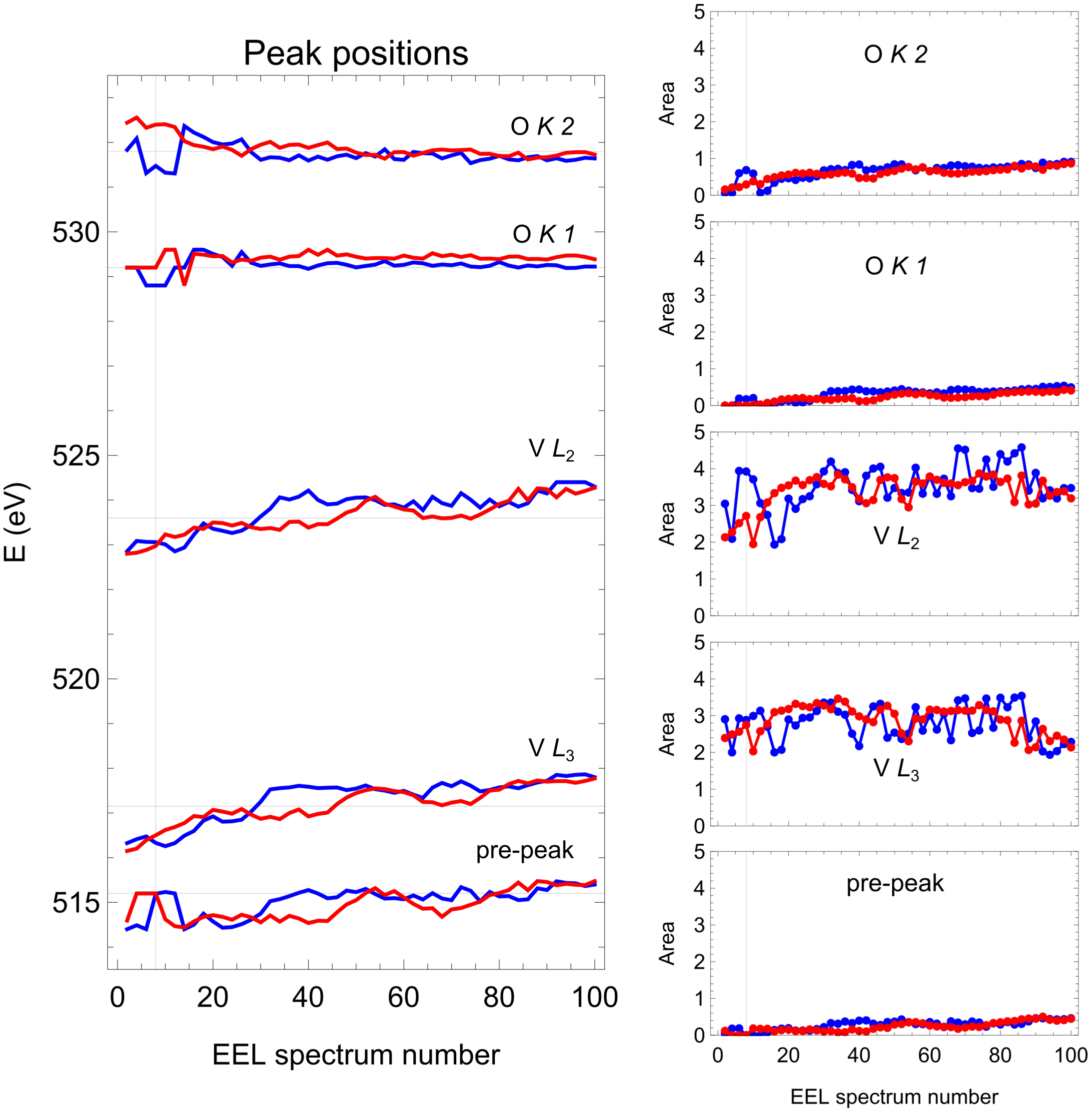

FIG. S5. Positions (energies) and areas of selected Gaussian peaks fitted to the spectra from Fig. S4. Blue and red points correspond to spectra acquired at room and high temperature, respectively.

S5. RAW LOW-LOSS EELS DATA

In Fig. S6 we show the low-loss EEL spectra as acquired after averaging 100 spectra from the area of interest marked by the small green square in Fig. 2(a) of the main text. In (a) we show entire spectra dominated by ZLPs, which have slightly different shapes due to a slight change in the microscope-spectrometer alignment during the time of the measurement. (b) A detail of the spectral region of interest. The signal plotted in Fig. 4(b) of the main text is determined as the difference between the raw signal (solid lines) and the fitted background due to the tails of the ZLPs (dashed lines). Further details on the acquisition of the spectra can be found in the Experimental Section of the main text.

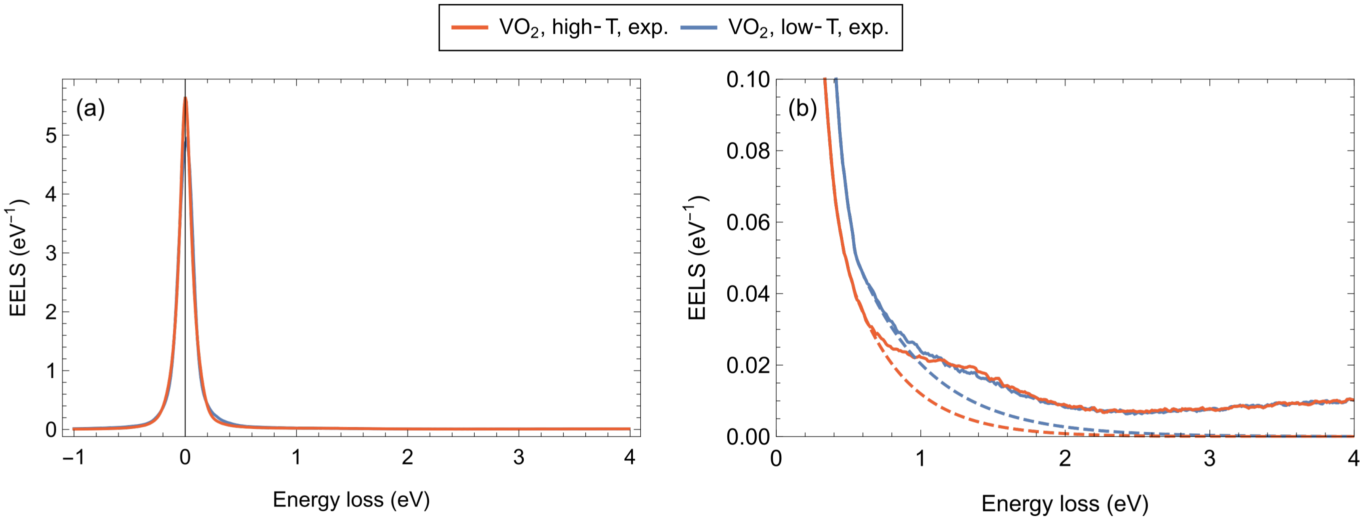

FIG. S6. Raw low-loss EELS data from the experiment. (a) Overview of the spectra obtained at 25 °C (blue line) and 150 °C (red line) dominated by the ZLPs. (b) Detail in the region where the signal of interest appears. Dashed lines follow the fitted background used for subtraction.

S6. COMPUTATIONAL DETAILS OF ELNES CALCULATIONS

The band structure calculations were performed with the all-electron WIEN2k code [1] employing the Strongly Constrained and Appropriately Normed (SCAN) meta-GGA functional [2], which, in contrast to the PBE functional, opens the band gap in the monoclinic VO_2 phase while it keeps the rutile VO_2 phase metallic [3]. We used the SCAN-optimized monoclinic and rutile VO_2 structures from Ref. [3] to calculate electronic structures and to model ELNES spectra of both phases. The paramagnetic nature of the rutile phase is approximated by an antiferromagnetic ordering along the rutile c axis. In order to build such magnetic ordering, we transformed the conventional rutile unit cell to the monoclinic-like superstructure according to Ref. [4]. Calculations of the monoclinic phase were performed in a non-spin polarized fashion, which reflects the diamagnetic nature of the monoclinic phase [5]. The muffin-tin radii for oxygen and for vanadium atoms were set to 1.72 Å and 1.88 Å, respectively in the rutile phase. Since the monoclinic phase contains much shorter O-V bonds, the radii were decreased to 1.57 Å and 1.73 Å for oxygen and vanadium, respectively, to prevent an overlap of muffin-tin spheres. The RKmax value was set to 8.0 and the tetrahedron smearing was considered in all calculations. The Brillouin zone was sampled with 170 k-points and the SCF cycle was converged to 10^{-6} Ry.

ELNES simulations were performed in the fully relativistic fashion employing the TELNES3 package incorporated in WIEN2k. The beam energy was set to 300 keV, collection semiangle and convergence semiangle were set to 12 mrad and 8.14 mrad, respectively, and the spectrometer broadening was set to 0.5 eV. The instrumental broadening was modelled by an additional smearing with a Gaussian of 1 eV FWHM. The core hole life time was modelled by a Lorentzian of 0.156 eV FWHM and excited state life time by a linear energy-dependent Lorentzian of width 0.1 E with E being energy above the Fermi level. The calculations were averaged over different lattice orientations as the regions of interest contain multiple grains across the slab thickness.

S7. COMPARISON OF EDX AND EELS

Our methodology comprises EDX and EELS, which are both related to the same excitation in the examined sample – the interband transition between the core electrons and valence electrons. However, the information retrieved from both methods differs due to a significant difference in their spectral resolution, which is around 120 eV for EDX and below 1 eV for EELS. EDX provides the elemental composition of the sample, while EELS captures fine details of the absorption edge related to the valence band profiles, which are specific to individual chemical compounds. We note that EELS could be in principle utilized to determine the elemental composition just as EDX, but in our study, we set a high spectral resolution at the price of a narrow spectral range insufficient for the elemental analysis.

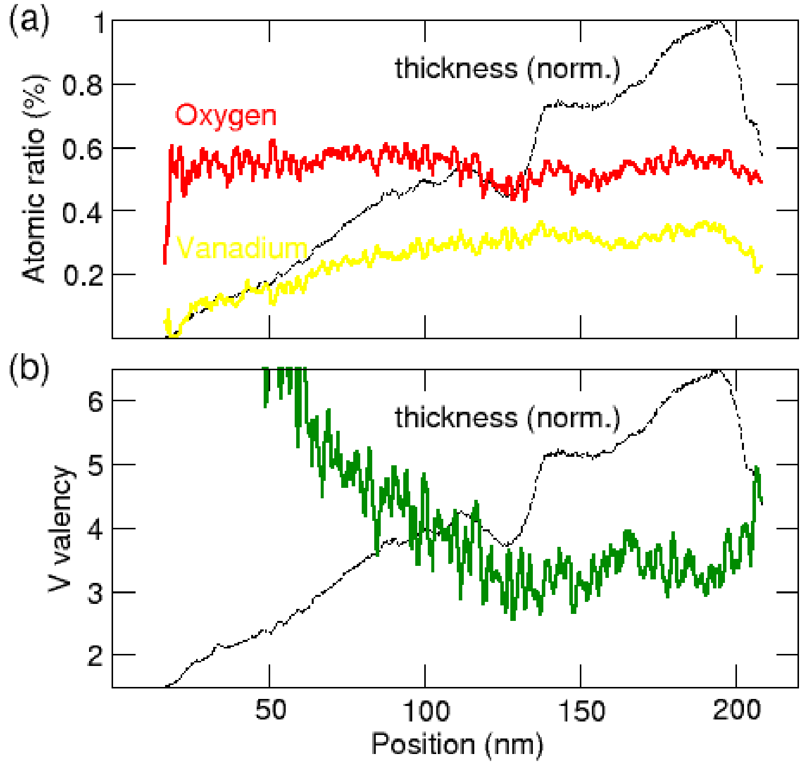

FIG. S7. (a) The elemental rate of vanadium (yellow line) and oxygen (red line) along the line scan of the lamella "IS" determined from EDX. (b) The effective valency of vanadium (green line) determined as the ratio of the elemental rates of oxygen and vanadium multiplied by 2. The value of 4 corresponds to VO_2, and the value above 5 indicates a probable presence of surplus oxygen. The relative local thickness of the lamella (determined from the HAADF image contrast) is shown by the black dashed line in both panels.

Figure S7 shows the average elemental composition of the lamella "IS". We observe that a relative amount of the oxygen (normalized to the amount of vanadium) is larger for the thinner parts of the lamella. Since the core-loss EELS analysis shows a decreased oxidation number of vanadium at the thinner parts, we infer a presence of surplus oxygen, either adatoms or as a part of oxidized contamination. The presence of surplus oxygen is also manifested on the core-loss EEL spectra assigned to vanadium monoxide (VO) at the low temperature, which show oxygen peaks unrelated to vanadium oxides in the energy range 535–545 eV [Fig. 3(a), spectrum labelled as VO+O].

Surplus oxygen was indirectly observed also in the porous parts of the lamella "S", with the mean stoichiometry $VO_{2.6\pm0.6)}$ (or $V_2O_{5.1\pm1.1}$), vanadium atomic rate of $(28 \pm 5)\,\%$, and oxygen atomic rate of $(72 \pm 9\,\%)$. Again, the core-loss EELS analysis of the pores on the lamella "IS" did not show any stoichiometry differences of vanadium oxide between the pores and the homogeneous part of the lamella, and we, therefore, attribute the surplus oxygen to the

increased contamination in the pores rather than increased oxidation number of vanadium (e.g. in V_2O_5).

[1] P. Blaha, K. Schwarz, F. Tran, R. Laskowski, G. K. H. Madsen, and L. D. Marks, WIEN2k: An APW+lo program for calculating the properties of solids, The Journal of Chemical Physics **152**, 074101 (2020).
[2] J. Sun, A. Ruzsinszky, and J. Perdew, Strongly Constrained and Appropriately Normed Semilocal Density Functional, Physical Review Letters **115**, 036402 (2015).
[3] J. Planer, Surface and Bulk Characterization of TransitionMetal Oxides: A Density Functional Theory Study of ZrO_2 and VO_2, Ph.D. thesis, TU Wien (2021).
[4] V. Eyert, The metal-insulator transitions of VO_2 : A band theoretical approach, Annalen der Physik **514**, 650 (2002).
[5] Y. Wang, X. Xi, Z. Li, E. Liu, Y. Yu, Y. Wu, G. Wu, and W. Wang, Dynamic signature of orbital selective Mott transition in the metallic phase of VO_2, New Journal of Physics **20**, 073026 (2018).
